# In vivo toxicity of bioreactor-grown biomass and exopolysaccharides from Malaysian tiger milk mushroom mycelium for potential future health applications

**DOI:** 10.1038/s41598-021-02486-7

**Published:** 2021-11-29

**Authors:** Siti Rokhiyah Ahmad Usuldin, Wan Abd Al Qadr Imad Wan-Mohtar, Zul Ilham, Adi Ainurzaman Jamaludin, Nur Raihan Abdullah, Neil Rowan

**Affiliations:** 1 Agro-Biotechnology Institute, Malaysia (ABI), National Institutes of Biotechnology Malaysia (NIMB), c/o HQ MARDI, 43400 Serdang, Selangor Malaysia; 2 Functional Omics and Bioprocess Development Laboratory, Institute of Biological Sciences, Faculty of Science, Universiti Malaya, 50603 Kuala Lumpur, Malaysia; 3 Bioresources and Bioprocessing Research Group, Institute of Biological Sciences, Faculty of Sciences, Universiti Malaya, 50603 Kuala Lumpur, Malaysia; 4 Environmental Science and Management Program, Institute of Biological Sciences, Faculty of Science, Universiti Malaya, 50603 Kuala Lumpur, Malaysia; 5 Department of Biotechnology, Kulliyyah of Science, International Islamic University Malaysia, 25200 Kuantan, Pahang, Malaysia; 6 Bioscience Research Institute, Athlone Institute of Technology, Athlone, Ireland; 7 Empower Eco Innovation Hub, Boora, Co. Offaly, Ireland

**Keywords:** Biological techniques, Biotechnology

## Abstract

Natural mycelial biomass (MB) and exopolysaccharides (EPS) of Malaysian tiger milk mushroom *Lignosus rhinocerus* are considered high-end components due to their high commercial potential value in drug discovery. This study aims to evaluate the toxicity of the mushroom extracts’ generated in a bioreactor using the zebrafish embryo toxicity (ZFET) model assay as a new therapy for treating asthma. Both MB and EPS extracts, at concentrations 0.16–10 mg/mL, were tested for ZFET and early development effects on Zebrafish Embryos (ZE) during 24–120 h post-fertilisation (HPF). Findings revealed that MB was deemed safe with an LC_50_ of 0.77 mg/mL; the EPS were non-toxic (LC_50_ of 0.41 mg/mL). Neither MB nor EPS delayed hatching nor teratogenic defects in the treated ZE at a 2.5 mg/mL dose. There were no significant changes in the ZE heart rate after treatments with MB (130 beats/min) and EPS (140 beats/min), compared to that of normal ZE (120–180 beats/min). Mixing both natural compounds MB and EPS did not affect toxicity using ZFET testing; thus, intimating their safe future use as therapeutic interventions. This represents the first study to have used the ZFET assay on MB and EPS extracts of *L. rhinocerus* for future health applications.

## Introduction

Mushrooms are filamentous fungi that have been used worldwide since prehistoric times^1,2^, and medically since at least 3000 BCE^3,4^. Mushrooms are heterotrophic organisms and have become increasingly important in research and industry^5^; food, medicines, cosmetics, detergents, and biofuels are examples of high-value products manufactured from fungi^6,7^. Furthermore, mushroom-derived extracts are becoming increasingly popular due to their potential usage in a wide range of vital health applications^8,9^. A focused interest in biorefining these products, such as via Green New Deal innovations from food waste streams, reflects a stronger emphasis on expanding ‘circularity’ and bioeconomy^10–14^. Mushrooms are classified in the kingdom of Fungi and have many active constituents, including, but possibly not limited to, polysaccharides, polysaccharide peptides, proteins, terpenoids, and nucleotides^15^. The most studied and used medicinally active ingredient in mushrooms is β-glucan. Previous research has revealed that β-glucans have broad metabolic and gastro-intestinal effects, including modulating the gut microbiome, altering lipid and glucose metabolism, and reducing cholesterol; thus, leading to the use of β-glucan as potential therapy for treating metabolic syndrome, obesity and diet regulation, gastrointestinal conditions, and to reduce the risk of cardiovascular and diabetes^16,17^.

The bioactive extracts derived from mushrooms can modulate the immune response affecting hematopoietic stem cells, lymphocytes, macrophages, T cells, dendritic cells, and natural killer cells. Murphy et al.^18^ reviewed over 200 patents that highlighted the therapeutic potential of β-glucans; this is evidenced by the fact that two glucans were licensed in Japan as immune-adjuvant therapy for treating cancer. Moreover, the pronounced immune-modulatory effects of β-glucans^18,19^ promoted their usage as adjuvant agents for treating cancers, immune-mediated conditions, rhinitis, respiratory infections, and to enhance wound healing^16,20^. However, further clinical testing and translation of β-glucans face significant challenges due to differences in source and extraction procedures^16^. We recently identified the active ingredients of *Lignosus rhinocerus* using 2D NMR analyses and reported on the antioxidative potential of (1,3)-β-D-glucan as an essential constituent^21^. However, many other compounds extracted from medicinal mushrooms have yet to be named, which are often referred to by gel chromatography fraction^15^; thus, highlighting the need to conduct and report on their safety.

The tiger milk mushroom, *Lignosus rhinocerus*, belongs to the Basidiomycota section of the Polyporaceae family and is classified as a filamentous fungus^22,23^. *L. rhinocerus* was grown in submerged-liquid fermentation (SLF) using a laboratory-scale stirred-tank bioreactor to achieve bulk cultivation and commensurate production of polysaccharides^21^. When compared to solid-state fermentation (SSF), SLF has several advantages, including limited space requirements, ease of scale-up, reliable and reproducible processing, ease of monitoring, and versatility^24^. Artificially cultivated *L. rhinocerus* is also an excellent replacement in developing therapeutic items. For example, exopolysaccharides (EPS) isolated from mushroom mycelial biomass (MB) have pharmacological properties as immunomodulatory, anti-inflammatory, antibacterial, antiviral, and antioxidant activities^25^. Chen et al.^26^ discovered that *L. rhinocerotis* mycelium grown in SLF does not cause mutagenicity or genotoxicity. The US Food and Drug Administration (FDA) standards, on the other hand, demand substantial proof of no hazard for commercial usage^26^.

Asthma affects 300 million individuals worldwide and is caused by a complex combination of inherited and environmental variables^23^. Allergic asthma is a long-term condition characterised by wheezing, shortness of breath, chest tightness, and coughing. In Malaysia, indigenous peoples have long used *L. rhinocerus* to treat asthma, while the majority of today's asthma medications are made up of steroids and other anti-inflammatory drugs^23^. Recent studies have reported on the efficacy of using EPS from medicinal mushrooms to ameliorate pro- and anti-inflammatory responses using ex vivo and in vivo infection models with therapeutic potential^16,18,27^. Aqueous extracts of *L. rhinocerotis* were reported to help reduce asthma-related variables in an asthma model^28^. In addition, a previous toxicity study indicated that feeding 1000 mg/kg of *L. rhinocerus* extract to rats had no detrimental consequences, hence it was considered safe^29^. As a result, more effective asthma treatment is required using *L. rhinocerus* as a helpful adjuvant or alternative to currently available asthma medications.

Zebrafish embryos have been extensively studied and documented as a reliable and popular model for developmental biology, toxicity, and, more recently, drug discovery^30^. Zebrafish may be readily bred, reared, and maintained in the laboratory^31^. Zebrafish embryos develop quickly, where they are fully developed five days after conception. Light microscopy can straightforwardly examine morphological structures and internal organs, such as the brain, eyes, heart, liver, and kidney due to the embryo's transparency. Dyes can be used to measure organ-specific and overall developmental toxicity visually or quantitatively. Due to its small size, a single Zebrafish embryo can be maintained in low fluid volumes for the first six days of development, including microtiter plates. The permeability of zebrafish embryos is prominent; for example, small chemicals added to fish water permeate the embryos, simplifying drug administration and assay processing^32^. Chemical screening can be completed after a few days due to the embryo's rapid development. The zebrafish is therefore a unique vertebrate model for high-throughput chemical screening, beneficial for pre-clinical drug discovery and toxicity assessment^33,34^.

A recent publication evaluating the toxicity of biomass-EPS comparable medicinal mushroom mycelial extracts revealed that the zebrafish embryo toxicity (ZFET) assay could be deployed as a safety screening approach before pre-clinical testing according to national and international standards^35^. Compared to human cell lines, research on the ZFET model is quick, resilient, efficient, and cost-effective for early development investigations; it also represents relevant genetic structure and equivalent critical organs and tissues^36,37^. Thus, this study aims to determine the toxicity of mushroom extracts using ZFET before they are developed and potentially commercialised as a new therapeutic intervention. To the best of our knowledge, there have been no toxicity studies using the ZFET model describing the use of MB and EPS of *L. rhinocerus* generated in the bioreactor.

As a result, this constitutes the second study to determine the toxicity levels of extracted MB and EPS from *L. rhinocerus* using a ZFET model to ensure product safety throughout the pre-commercialisation phase. Notably, the present rare *L. rhinocerus* strain ABI (Agro-Biotechnology Institute Malaysia) was successfully isolated and identified from a tropical forest near Lata Iskandar, Pahang, Malaysia^21^; however, limited information has been published on its therapeutic potential. This study reports on the use of ZFET assay on bioreactor-grown Malaysian medicinal mushroom *L. rhinocerus* MB and EPS extracts. This study explicitly addresses LC_50,_ embryonic hatching delays, teratogenic defect, and heart rate response with clear microscopic images. Furthermore, these findings also support the possibility of future pre-clinical trials involving the safe use of MB and EPS for prospective health applications in respiratory diseases.

## Results

### Zebrafish embryo survival rate after MB and EPS exposure

**Figure 1:**
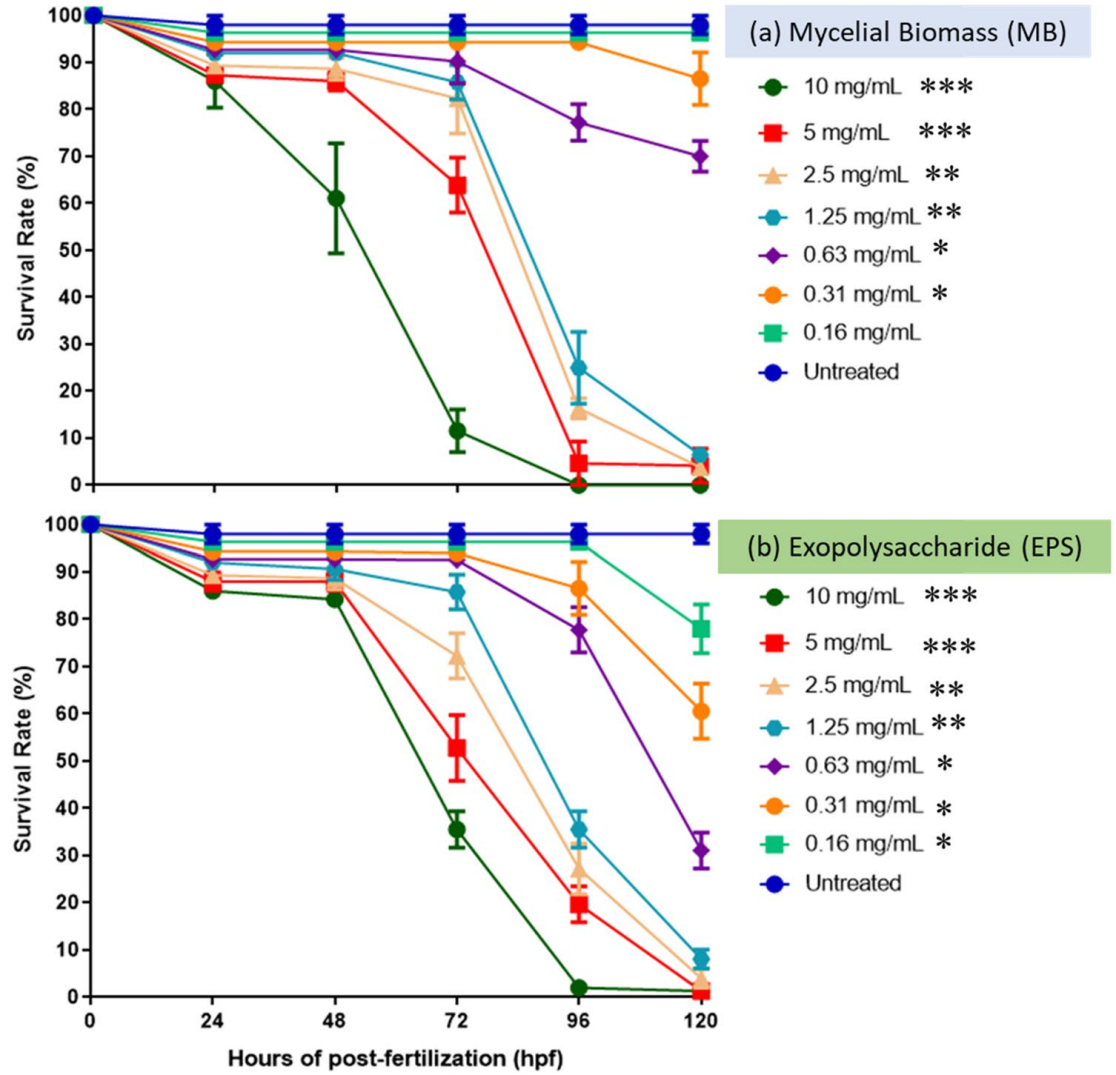
The performance of Tiger milk mushroom, *Lignosus rhinocerus* strain ABI (**a**) MB and (**b**) EPS extract at concentrations of 0.16–10 mg/mL on the survival rate of zebrafish embryos at 0–120 h. Symbols: ***p < 0.001, **p < 0.01 and *p < 0.05. No embryos survived for both samples at concentration tested > 5.0 mg/mL after 96 h-post-fertilization (HPF).

The survival rate of zebrafish embryos following MB and EPS exposure was studied between 0 and 120 h at MB and EPS extract concentrations of 0.16–10 mg/mL. The study period included larvae as the zebrafish embryos hatch typically 48 to 72 h post-fertilisation (HPF). The survival rate of untreated embryos, between 0 and 120 HPF, was 100% (Fig. 1a). At 48 HPF, the survival percentage for embryos treated with MB fell to 85% and 60% at > 5 mg/mL and 10 mg/mL, respectively. At 72 HPF, the survival rate declined to 80%, 65%, and 10% at < 2.5 mg/mL, 5 mg/mL, and > 10 mg/mL, respectively. At concentrations > 1.25 mg/mL, the survival rate at 96 HPF was 20%, and after 120 HPF; it was observed that no embryos survived at concentrations > 1.25 mg/mL (Fig. 1a). The survival rate of embryos (prior to hatching) and larvae (post-hatching) treated with EPS (0.110 mg/mL) during the five days is shown in Fig. 1b. Between 0 and 120 h of HPF, untreated embryos (control) exhibited a 100% survival rate. After 72 h of HPF exposure, the survival rate declined to 90%, 85%, and 50% at a concentration of 0.63 mg/mL, 1.25 mg/mL, and 5 mg/mL, respectively. At 96 HPF, the survival rate declined to 75% at concentrations < 0.63 mg/mL and 30% at concentrations > 1.25 mg/mL. At 120 HPF, survival rates at concentrations 0.63 mg/mL declined to 30%, while survival rates at concentrations > 1.25 mg/mL were 0%, with no surviving embryos. Overall, the results suggest that MB and EPS extracts delay hatching at doses < 1.25 mg/mL.

### Zebrafish embryos mortality after MB and EPS exposure

**Figure 2:**
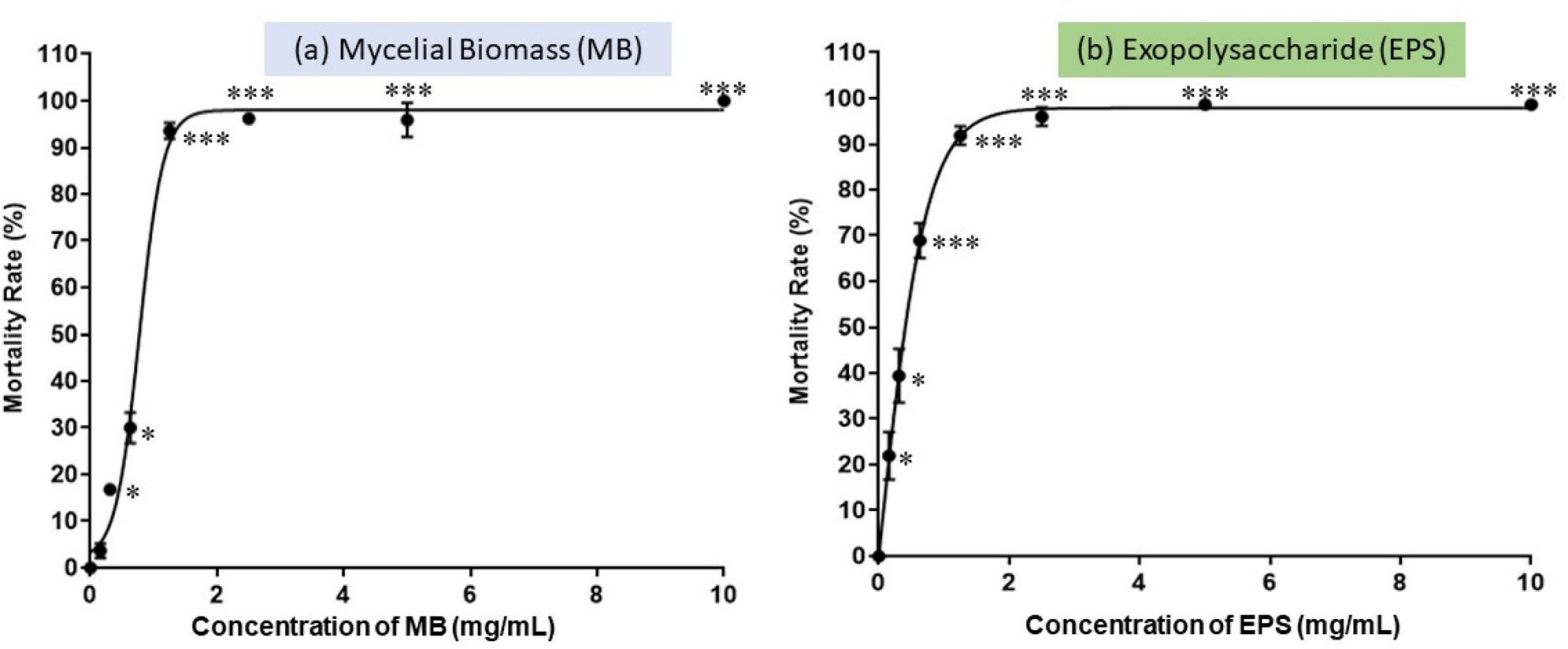
Effect of Tiger milk mushroom, *Lignosus rhinocerus* strain ABI (**a**) MB extract at concentrations of 0.16–10 mg/mL and (**b**) EPS at concentrations of 0.01–10 mg/mL on zebrafish embryos mortality rate after 120 HPF. Symbols: ***p < 0.001, **p < 0.01 and *p < 0.05. The LC_50_ value for MB extract was 0.77 mg/mL while LC_50_ value for EPS extract was 0.41 mg/mL.

Overall, MB and EPS extracts had dose- and time-dependent fatal effects. Figure 2 shows a high survival rate (90%) of zebrafish embryos at concentrations of MB and EPS extracts < 1.25 mg/mL. Both MB and EPS extracts had a low survival rate at high concentrations (> 1.25 mg/mL), and none survived after 96 HPF. As a result, the fatal concentration for 50% (LC_50_ value) of zebrafish embryos exposed to MB was 0.77 mg/mL, while the LC_50_ value of the EPS extract was 0.41 mg/mL.

### Zebrafish embryos hatching after MB and EPS exposure

**Figure 3:**
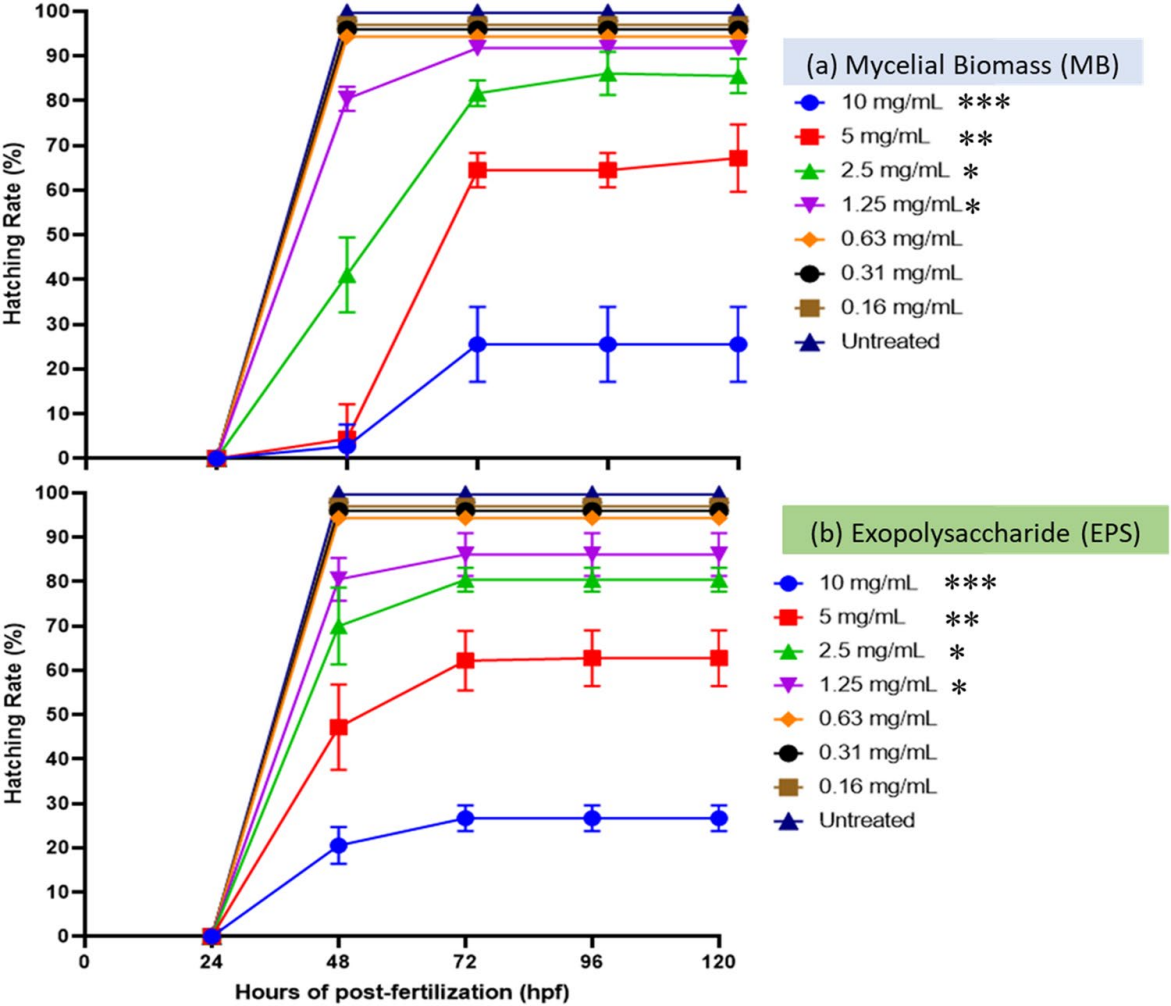
Hatching rate of zebrafish embryos at 0 to 120 HPF with Tiger milk mushroom *Lignosus rhinocerus* strain ABI, with MB and EPS extract at concentrations of 0.16–10 mg/mL. Symbols: ***p < 0.001, **p < 0.01 and *p < 0.05. (**a**) For MB, a low hatching rate (< 25%) was observed at concentration 10.0 mg/mL due to a high mortality rate. Meanwhile, (**b**) for EPS, a low hatching rate (< 30%) was observed at concentrations of 10 mg/mL due to a high mortality rate. High hatching rate was observed at concentrations > 1.25 mg/mL (> 80%).

Based on the embryo observations, increasing the mushroom extract concentrations can decrease the percentage hatchability. Figure 3a illustrates the hatching rate of zebrafish embryos treated with MB and EPS (both at 0.1610 mg/mL) at 0–120 HPF. No significant changes in the hatching rate were found when the zebrafish embryos were treated with MB extract at a 0.63 mg/mL concentration. However, at 48 HPF, the rate declined to 80% at concentrations > 1.25 mg/mL. At 72 HPF, the hatching rate was lowered to 65% at 5 mg/mL. Further reduction was observed (25% hatching rate) when treated with 10 mg/mL MB, implying a high death rate after 72 HPF. The hatching rate of EPS did not alter significantly after the treatment with 0.63 mg/mL MB. Less than 85% of the embryos hatched were observed after a 48-h treatment with EPS at > 1.25 mg/mL. However, due to a significant mortality rate at 72 HPF, zebrafish larvae treated with EPS at 10 mg/mL doses had the lowest hatching rate (30%).

### Zebrafish embryos heart rate after MB and EPS exposure

**Figure 4:**
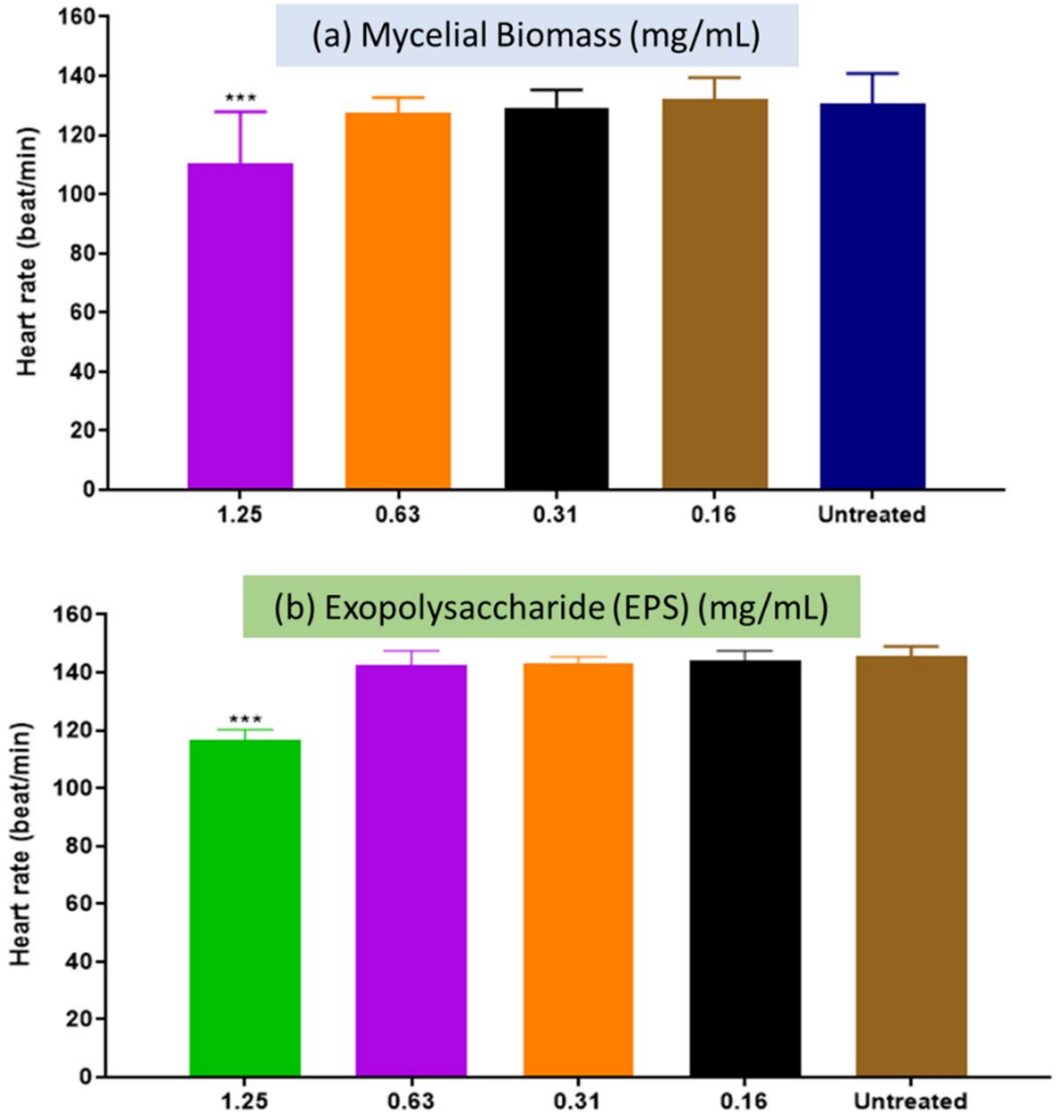
Effect of Tiger milk mushroom *Lignosus rhinocerus* strain ABI, with MB and EPS extract at concentrations of 0.16–10 mg/mL on the heart rate of zebrafish embryos at 96 HPF. ***p < 0.05 significantly different from the untreated group (zebrafish embryos in medium only). *P < 0.05 significantly different from the untreated group (zebrafish embryos in medium only). For (**a**) MB, no data at concentrations > 2.5 mg/mL due to embryo death. Meanwhile, (**b**) for EPS, no data at concentrations > 2.5 mg/mL was recorded due to embryo death.

During the development of many model species, including zebrafish, the heart is the major functioning organ^38^. Previous research has shown that the average heart rate of zebrafish embryos is 120–180 bpm, which is much closer to that of humans^39^. As shown in Fig. 4, the heart rates of zebrafish larvae at 96 HPF (4 days) for both the MB (Fig. 4a) and EPS (Fig. 4b) treatments were 130 and 140 bpm, respectively. Both extracts exhibited no significant difference in the heart rate of zebrafish larvae at 96 HPF at lower concentrations (relative to higher doses in Fig. 3), ranging between 0.161.25 mg/mL for MB and 0.161.25 mg/mL for EPS. The heart rate of zebrafish larvae at these concentrations was not determined because both MB and EPS extracts at 2.5, 5, and 10 mg/mL demonstrated very little to no survival at 96 HPF.

### Morphology of the larvae and zebrafish embryos after MB and EPS exposure

**Figure 5:**
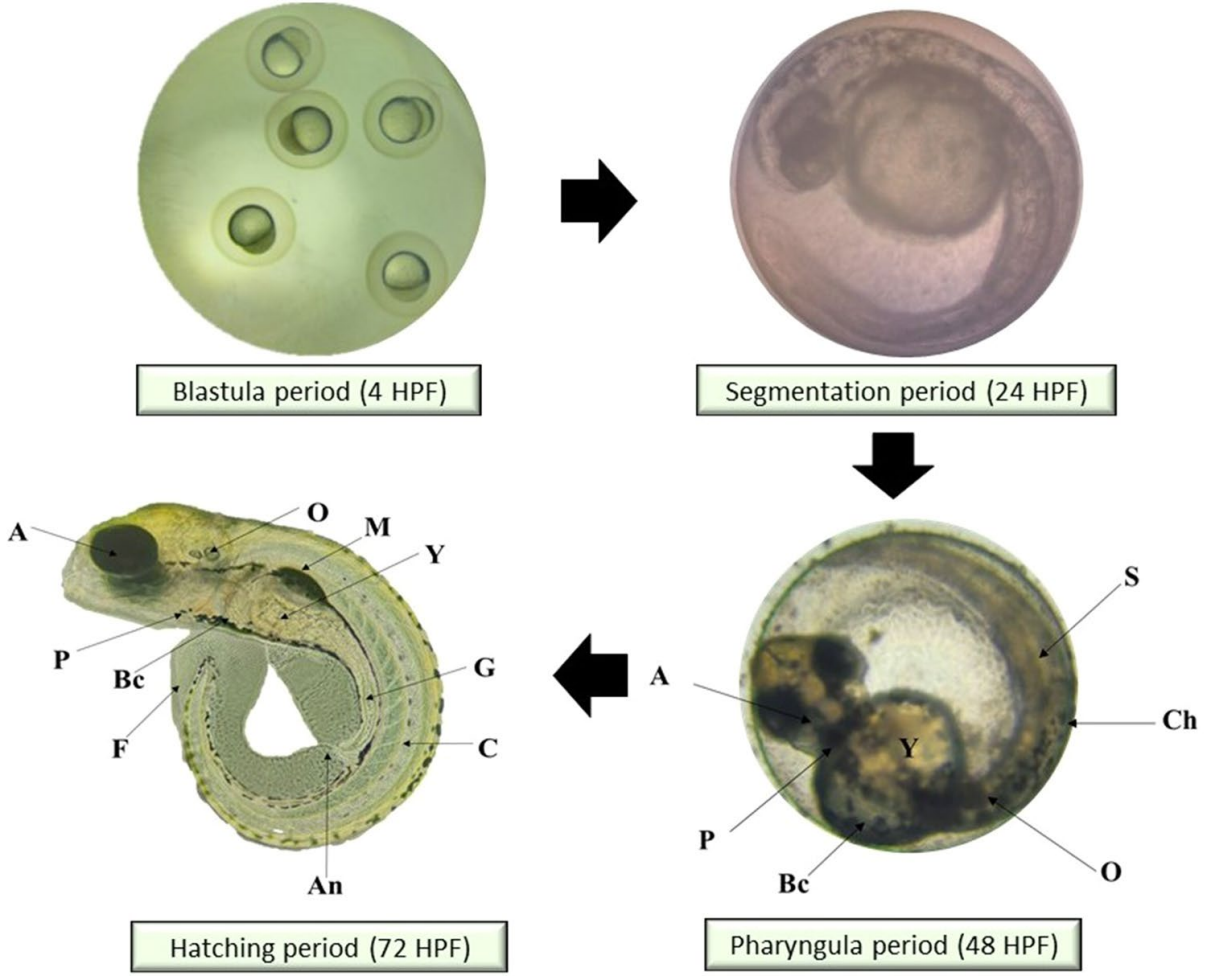
Effect of MB-EPS extracts (0.16–10 mg/mL) of Tiger Milk mushroom *Lignosus rhinocerus* strain ABI showing normal zebrafish embryogenesis at different HPF development. There were four periods depicted as according to Taufek et al.^16^: (**a**) Blastula (4 HPF), (**b**) Segmentation (24 HPF), (**c**) Pharyngula (48 HPF), and (**d**) Hatching (72 HPF). A—eye anlage; An—anus; Bc—blood cells; C—chorda; Ch—chorion; F—fin; G—gut; M—melanophores; O—ear bud; P—pericardium; S—somites; Y—yolk sac. Scale bar = 0.5 mm. The inverted microscope procedure was used to produce the images.

**Figure 6:**
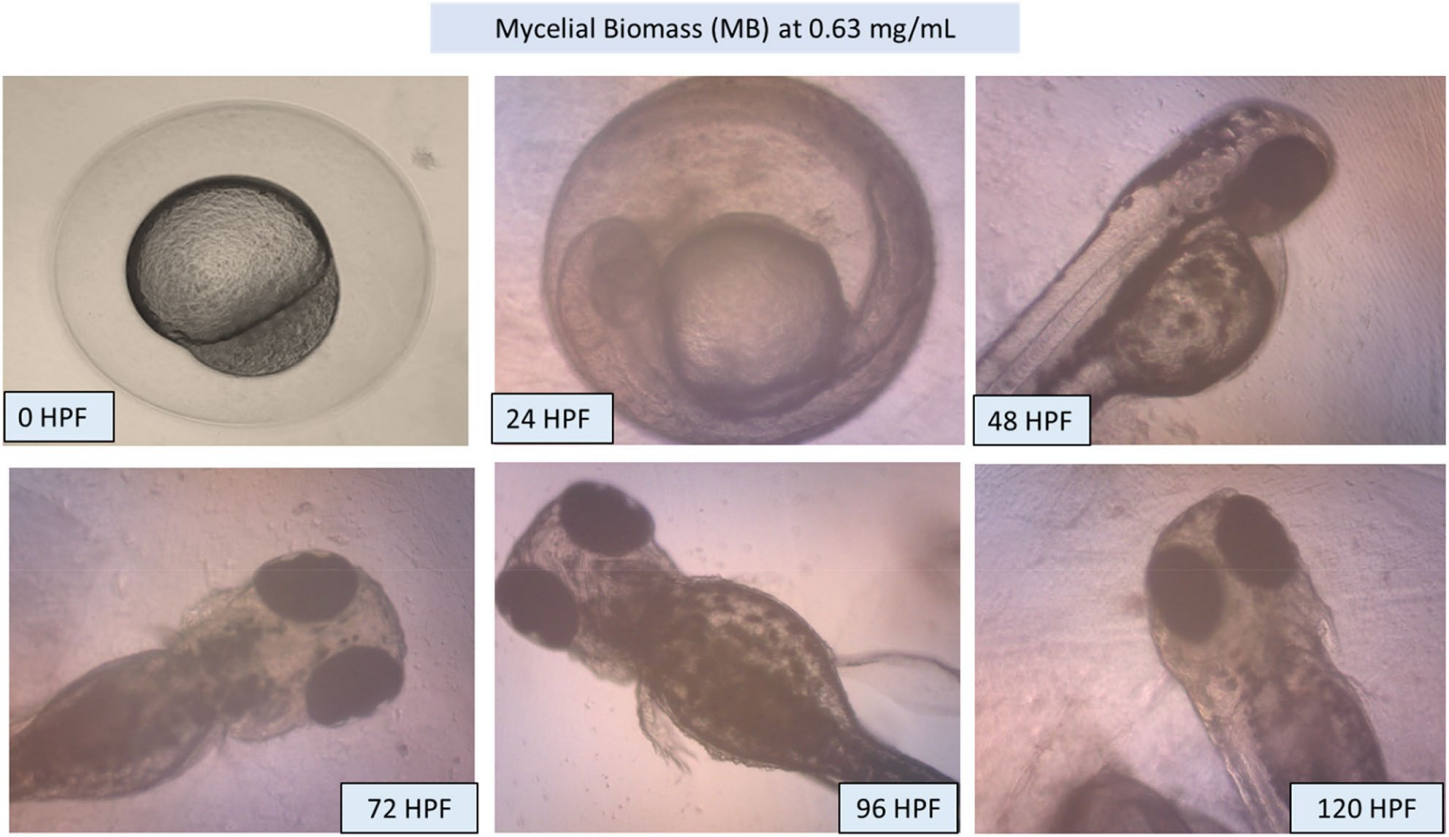
Illustrations of zebrafish embryo and larvae development after treated with Tiger Milk mushroom *Lignosus rhinocerus* strain ABI at EPS concentration of 0.63 mg/mL. Descriptions were captured using an inverted microscope at 100X (0 and 24 HPF) and 40X magnification (48–20 HPF).

**Figure 7:**
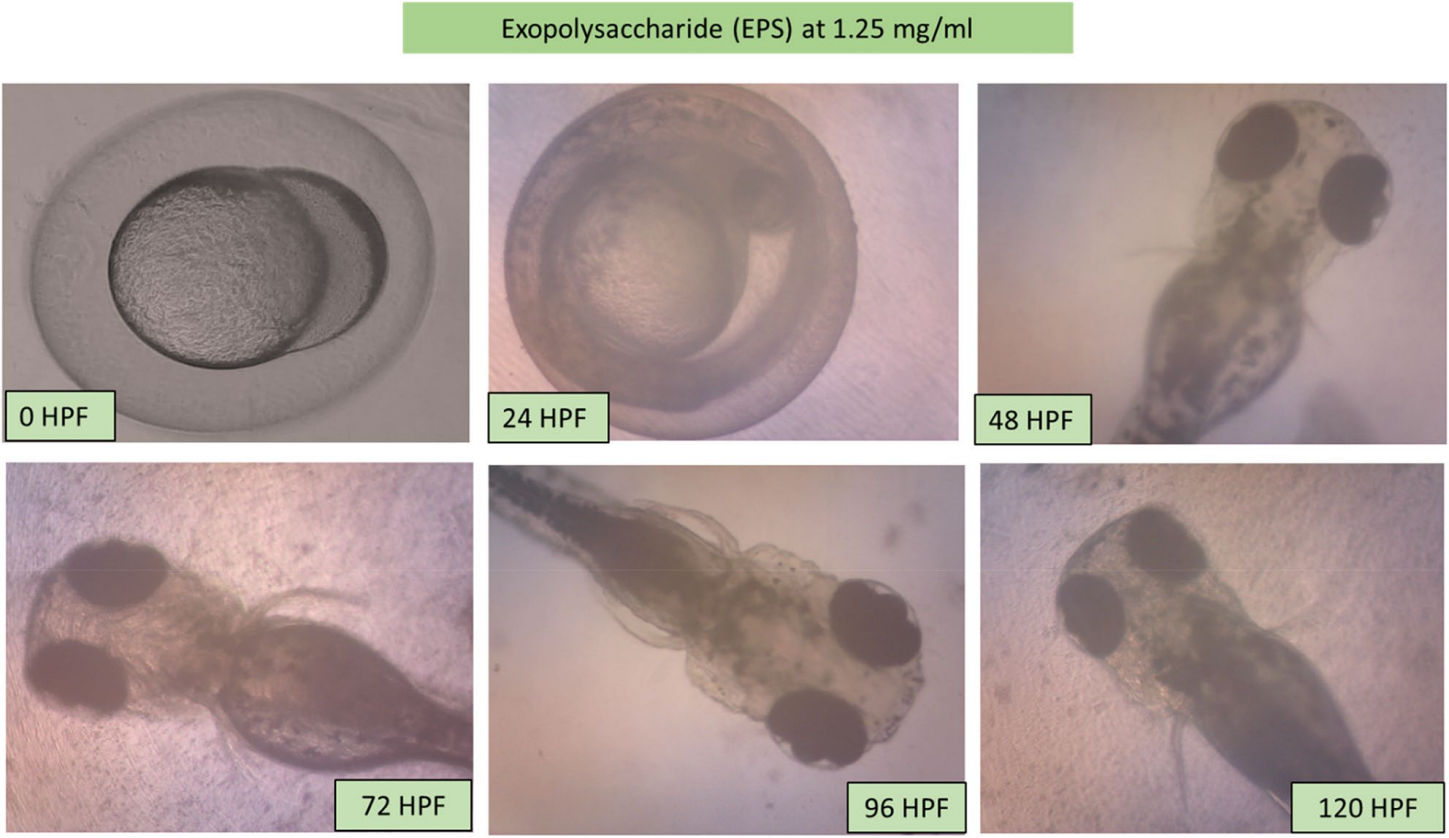
Illustrations of zebrafish embryo and larvae development after treated with Tiger Milk mushroom *Lignosus rhinocerus* strain ABI at EPS concentration of 1.25 mg/mL. Descriptions were captured using an inverted microscope at 100X (0 and 24 HPF) and 40X magnification (48–20 HPF).

**Figure 8:**
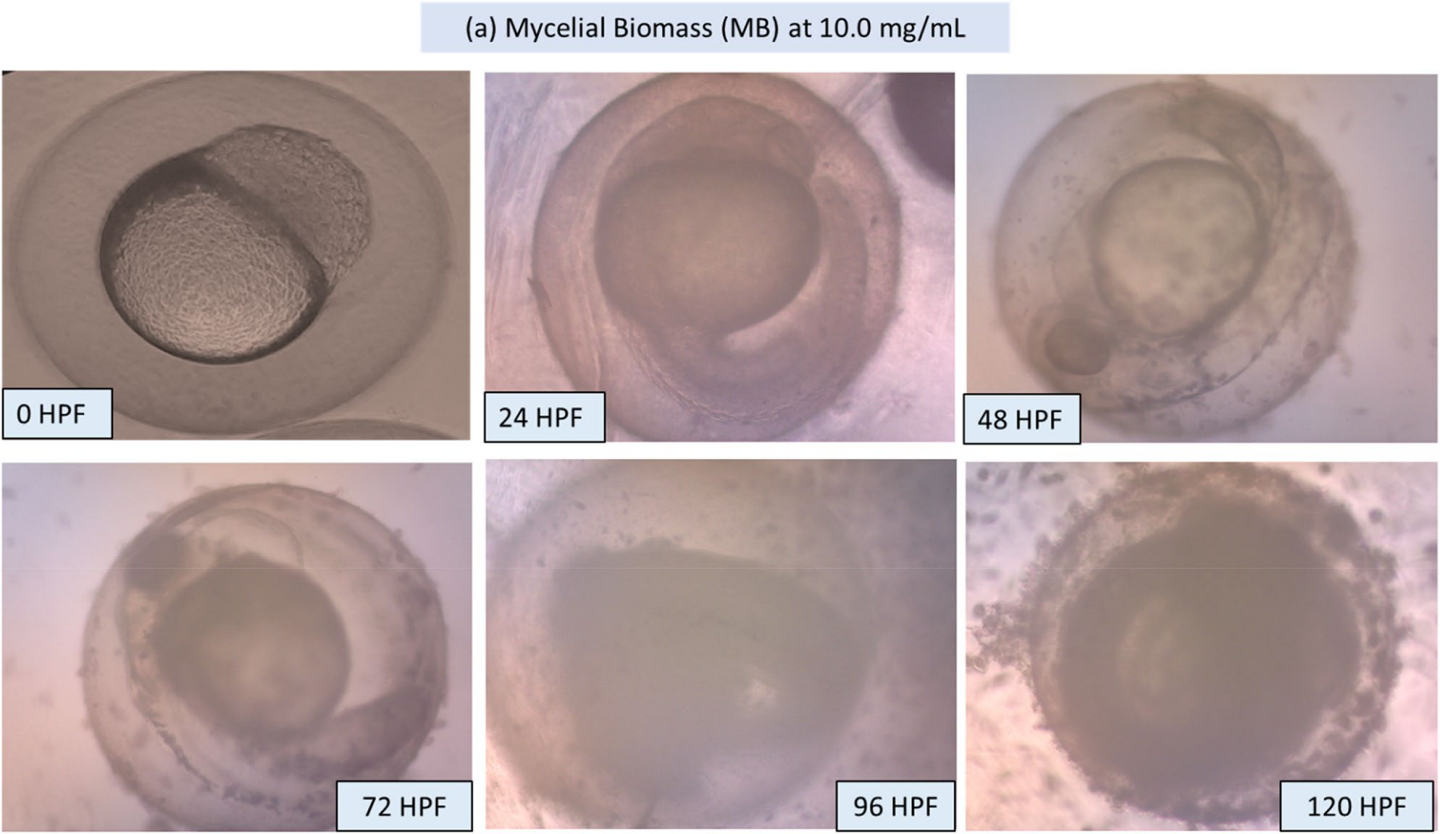
Illustrations of zebrafish embryo and larvae development after treated with Tiger Milk mushroom *Lignosus rhinocerus* strain ABI at high EPS concentration of 10.0 mg/mL. Descriptions were captured using inverted microscope at 100X (0 and 24 HPF) and 40X magnification (48–20 HPF).

**Figure 9:**
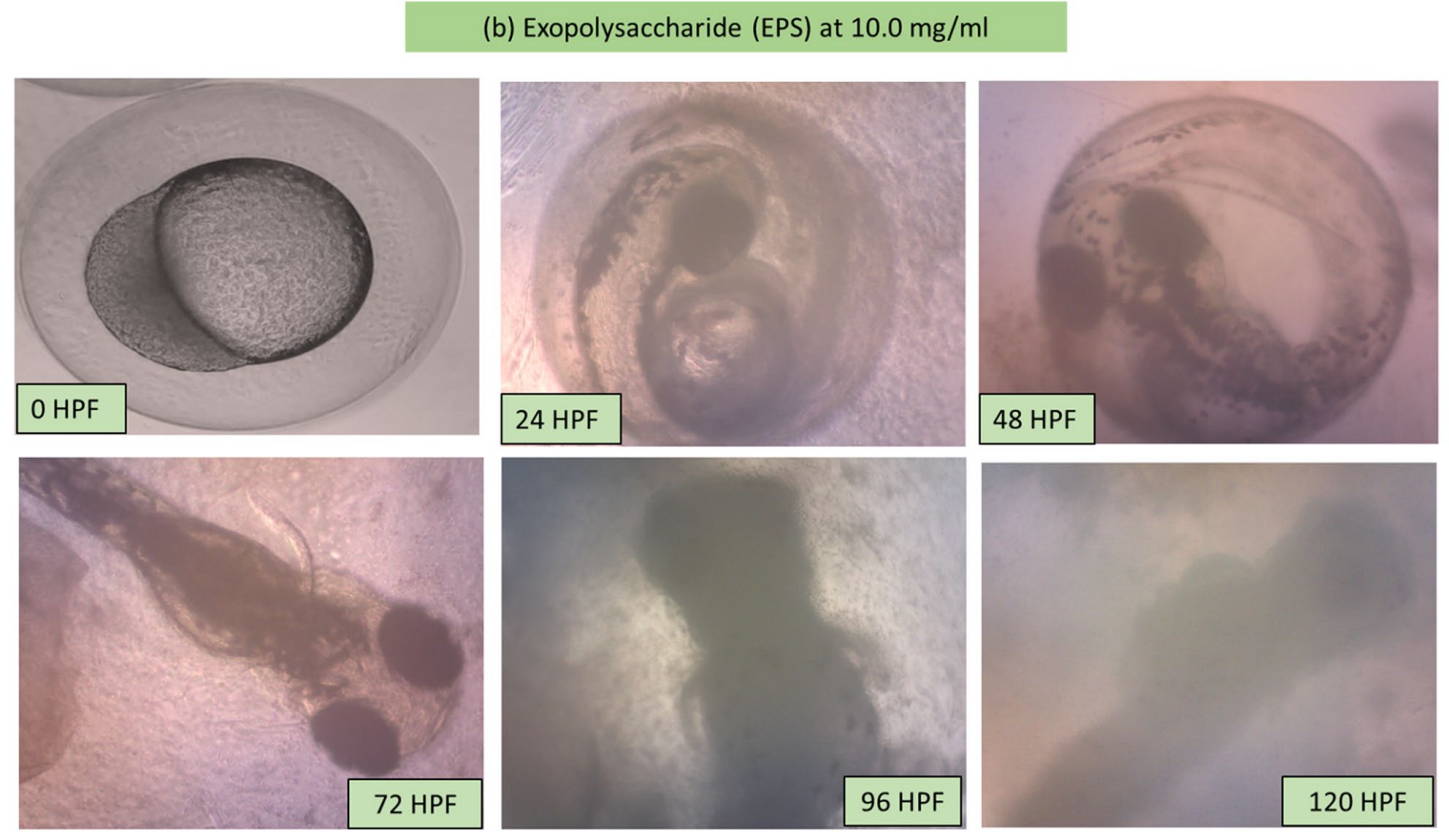
Illustrations of zebrafish embryo and larvae development after treated with Tiger Milk *Lignosus rhinocerus* strain ABI at high EPS concentration of 10.0 mg/mL. Descriptions were captured using an inverted microscope at 100X (0 and 24 HPF) and 40X magnification (48–20 HPF).

Potential morphological abnormalities in embryos and larvae were measured from 0 to 120 HPF. There was no apparent teratogenic effect on embryos and larvae after 120 h of exposure to MB and EPS at 0.63 mg/mL and 1.25 mg/mL, respectively (Fig. 5). These findings infer that MB and EPS have no teratogenic effects on zebrafish embryo development prior to- and post-hatching**.** The unaffected development of zebrafish embryos and larvae after exposure to 0.63 mg/mL MB and 1.25 mg/mL EPS are shown in Fig. 6 and Fig. 7; however, numerous defects were observed when the concentration of MB and EPS increased to 10 mg/mL (Fig. 8 and Fig. 9). Coagulated embryos observed between 24 HPF (segmentation) and 48 HPF (pharyngula), along with the loss of yolk sac preventing hatching, were the most common abnormalities reported using MB treatments. Moreover, EPS-treated zebrafish hatched at 72 HPF, where tail deformity and damaged blood cells were observed after 120 HPF, with various defects included missing fins, guts, and melanophores.

## Discussion

*Lignosus rhinocerus* is well-known for its therapeutic values, particularly as potential treatment of respiratory diseases. Previous reports have highlighted that the sclerotia, mycelium, and exopolysaccharides of *L. rhinocerus* contain similar bioactive compounds to β-glucans^21^. Nowadays, β-glucans have gained appeal for several emerging applications, including biopolymers^40^ and biomedicines^41^. Notable potential therapeutic properties recently uncovered of mushroom-derived β-glucans include: (a) new or complementary immunotherapies against Coronavirus disease (SARS-CoV-2)^18^; (b) new therapeutic agent for mitigating diseases associated with gastrointestinal mucosal damage, such as peptic ulcers and inflammatory bowel disease^42^; (c) anticancer drugs for lung and breast cancer^43^; and (d) asthmatic treatment^28,44^. However, there is a substantial gap in knowledge surrounding the toxicity (if any) of these mushroom-derived bioactive compounds, particularly on MB-EPS extracts. Therefore, the use of Zebrafish trials could aid product development and implementation. Hence, this work investigated and reported on the acute toxicity of zebrafish embryos post exposure to MB and EPS derived from a rare Malaysian-origin Tiger Milk mushroom *L. rhinocerus* grown in a bioreactor.

The ZFET approach was used to expose fertilised zebrafish embryos to quantities of *L. rhinocerus* extract, MB (0.16–10 mg/mL), and EPS (0.16–10 mg/mL) shown to be non-toxic. Overall, both MB and EPS at 2.5 mg/mL concentrations did not delay embryo hatching and had a > 80% survival rate between 24 and 120 HPF. In addition, there were no significant differences in the embryo heart rate between the MB and EPS concentrations of 1.25 mg/mL. At MB and EPS doses of > 0.63 mg/mL and > 1.25 mg/mL, respectively, teratogenic effects were observed with evident zebrafish embryo defects. The test revealed that MB has a larger LC_50_ value of 0.77 mg/mL than EPS, with a lower LC_50_ value of 0.41 mg/mL. Although both MB and EPS extracts were obtained from *L. rhinocerus* mycelium, the compound composition may differ owing to the fruiting body and mycelial extraction procedures^45,46^. *L. rhinocerus* mycelium and culture broth demonstrated similar or increased bioactivities, including antioxidant capacities, compared to the use of fruiting bodies^45^. Moreover, EPS exhibited a lower LC_50_ value than MB did due to its different mycelial extraction methodology. This is possibly related to MB being directly obtained from dried fungal mycelium, whereas EPS is derived from post series of physicochemical extractions using active fungal mycelia^21,47^. The embryo's ability to burst through the chorion (Fig. 8) and hatch after five days may be limited by morphological defects such as tail deformity. A coagulated embryo and the absence of a heartbeat are both considered deadly.

Certain medicinal mushrooms have also been tested for their toxicity on zebrafish embryos in comparison to *Lignosus* species. Recent research on *Ganoderma lucidum* exposure found that MB did not affect ZE hatching at concentrations ranging from 250 to 5000 g/mL and EPS at 3000 g/mL. Notably, neither MB nor EPS were teratogenic at concentrations < 3000 g/mL^35^. Neither EPS or endopolysaccharide (ENS) concentrations of 1 mg/mL in *G. applanatum* cause embryo hatching delays. They were shown to have an 88% survival rate when tested from 24 to 120 HPF^48^. Consequently, this new ZFET data could be helpful in the identification of potential health risks associated with the MB-EPS consortia. However, more testing is merited to identify the LC_50_ value of MB-EPS extract for large-scale human trials and larger animals before this innovation may be used commercially (e.g. pigs, rabbits, and adult trout). A similar biosafety approach using zebrafish was used to test EPS from the wild-Serbian mushroom *G. applanatum*^49^, which exhibited a higher yet safe LC_50_ value (1.41 mg/mL) than that of the current wild-Malaysian *L. rhinocerus* study (0.41 mg/mL). The MB of *L. rhinocerus* demonstrated a harmless biosafety status of bioreactor cultivated *L. rhinocerus* mycelia and EPS products; thus, supporting further pre-commercialisation trials. Usuldin et al.^21^ found that MB production (~ 6 g/L: 30 g dry form) from a 5-L bioreactor culture supports high EPS yield, which can be produced in large quantities. When compared to dried polysaccharides, powdered MB is more applicable in the pharmaceutical industry. The latter is notable as 300 mg of dry tuber biomass from the Malaysian *L. rhinocerus* has been reported to potentially improve respiratory health in both in vivo and in vitro models^34^.

This study therefore constitutes the first toxicity investigation of *L. rhinocerus* grown in a bioreactor, with the results compared with that of extracts from other *Lignosus* species. Table 1 shows details of four studies assessing the effect of *L. rhinocerus* MB on cervical cancer cells (24 mg/mL)^50^, neurite bearing cells (1.75–5.93 mg/mL)^51^, MTT assay for normal human cells (200 μg/mL)^45^, and developmental toxicity in pregnant Sprague–Dawley (SD) rats (3.4 mg/mL)^52^. Notwithstanding this, there is no published research on the toxicity of EPS. The study results are significant where the Zebrafish 3.0 toxicity model was used to evaluate and assess what was to be non-toxic mycelial biomass (0.77 mg/mL) and EPS (0.41 mg/mL) in *L. rhinocerus* bioreactor samples. This Zebrafish model offered evidence that the use of Malaysian bioactive mycelial biomass and polysaccharides *L. rhinocerus* may be safe as a new therapeutic intervention.

**Table 1:** Similarity with literature for non-toxicity evaluation of mycelium biomass (MB) and exopolysaccharide (EPS) from the rare Tiger milk mushroom *Lignosus* sp. [NA: Not Available].

Fungal source	Toxicity model	Image	Non-toxic concentrations (mg/mL)		References
			Mycelial Biomass (MB)	Exopolysaccharide (EPS)	
*L. rhinocerus*	In vivo—Zebrafish embryos and larvae		0.77	0.41	*Current study*
*L. rhinocerotis*	In vitro—Cervical cancer cells (Ca Ski, HPV-16)	NA	25	–	^50^
*L. rhinocerotis*	In vitro—Differentiating mouse neuroblastoma (N2a) cells		1.75–5.93	–	^51^
*L. rhinocerotis*	In vitro-MTT assay	NA	0.2	–	^45^
*L. rhinocerotis*	In vivo—Developmental toxicity in pregnant Sprague–Dawley (SD) rats	NA	3.4	–	^52^

Furthermore, the findings from this research highlight the increasing trend towards the intensive yet sustainable exploitation of bio-based resources from food and marine ecosystems, from the emergence of the bioeconomy^11^. These bio-inspired materials may be refined and scaled up for commercial use through advances in biotechnology, as described here^49^. Notably, this emerging area will be future-proofed through accelerating digitalisation, where metadata outputs will potentially inform food for therapeutics, cosmetics, personal care products, and smart packaging, along with offering putative interventions to help mitigate the Covid-19 disease^10,51,53^.

## Conclusion

In conclusion, this is the first study on the use of ZFET assay on bioreactor-grown Malaysian medicinal tiger milk mushroom *L. rhinocerus* MB and EPS extracts. MB (LC_50_: 0.77 mg/mL) was harmless, whereas EPS (LC_50_: 0.41 mg/mL) are practically non-toxic. The ZFET assay offers a fast, affordable, robust, and efficient early development approach to evaluating extracts from medicinal fungi for future use as asthmatic medication. Specifically, this study provides evidence of the potential of *L. rhinocerus* as an alternative or adjuvant to the current drugs used for the management of respiratory diseases. Additionally, for the early medication development process, zebrafish can be utilised to quickly discover potentially dangerous chemicals and prioritise compounds for additional pre-clinical and clinical testing. The adaptation of conventional instruments in conjunction with new nanotechnology discoveries will help to further increase the use of zebrafish for drug screening.

## Methods

### Tiger milk mushroom

Wild Malaysian tiger milk mushroom, *L. rhinocerus* strain ABI was isolated from Lata Iskandar, Pahang, Malaysia, from the tropical rainforest (23 °C to 28 °C; 4.1949° N, 101.1923° E)^21^. The sclerotium was cultured on a potato dextrose agar (PDA) plate (Sigma-Aldrich, Dorset, UK) and incubated at 30 °C under dark conditions. The strain was stored and maintained on PDA slants at 4 °C^54^.

### Culture conditions

The fungal inoculum was prepared according to Wan Mohtar et al.^55^ blueprints fungal production plan, including two seed culture stages. The mycelium was cultivated for ten days under dark conditions at an initial pH of 5, 150 rpm, and 30 °C with slight adjustments for the first seed culture. Four mycelial agar squares (1 cm x 1 cm each) were cut from a ten-day-old plate culture and inoculated in a 250 mL Erlenmeyer flask using sterile scalpels (100 mL of medium). The first seed culture was then homogenised for 10 sec with a sterile Waring hand mixer to produce more hyphal tips with uniform mycelium diameters. The homogenised mycelial culture was transferred to a 500 mL shake flask (200 mL medium) as the inoculum for the second seed culture and incubated for 11 d under dark circumstances on an orbital shaker at initial pH 5, 150 rpm, and 30 °C. Unless otherwise stated, the liquid culture medium of seed cultures contained glucose (3% (w/ v), yeast extract (0.1% (w/ v), peptone (0.2% (w/ v), potassium dihydrogen phosphate (KH_2_PO_4_) (0.046% (w/ v), dipotassium hydrogen phosphate (K_2_HPO_4_) (0.1% (w/ v), and magnesium sulphate heptahydrate (MgSO4.7H).

### High-scale bioreactor fermentation

A stirred-tank (STR) bioreactor was used with a total volume of 5 L (3.5-L working volume) (Sartorius Stedim, Biostat B-plus, Germany). Blueprint of Usuldin et al.^21^ was followed; 10% (v/v) of the seed culture was used to inoculate the STR using parameters as follows: temperature (30 °C); pH 5.0; dissolved oxygen (DO) (20–40%); air flow rate 3 L/min; agitation speed (200 rpm). The mycelium was cultured in the bioreactor for 11 d and the resulting mycelial pellets were isolated. The media formulation for the bioreactor used was the same as that for the shake flask, unless otherwise stated.

### Mycelial biomass and exopolysaccharide production

The bioreactor's harvested mycelial biomass (MB) was filtered three times with distilled water using a vacuum Buchner funnel filter. The filtered MB was dried at 35 °C in a food dehydrator until it reached a consistent weight^47^. The filtrate was precipitated by adding 95% (v/ v) ethanol at a ratio of 1:4 to the filtrate and left overnight at 4 °C to obtain the EPS. After that, the sample was centrifuged for 15 min at 10,000 rpm. The supernatant was discarded, and the pellet was dried at 35 °C in a food dehydrator until it reached a constant weight.

### Sample preparation for the toxicity test

Dried MB and EPS were prepared at room temperature for toxicity testing. A 10 mg/mL of stock solution was prepared by dissolving dried MB and EPS in embryo media (Danio-SprintM media), which was then diluted two-fold and further in a 96-well microplate (200 µL/well) using serial dilutions to obtain seven different concentrations in the 0.16–10 mg/mL range. For a standard control, zebrafish embryos in embryo media solution were used as an untreated control sample (0 mg/mL).

### Upkeep and breeding of zebrafish system

A couple of adult zebrafish were placed in a breeding tank the day before the breeding occurred to set up the system. The following day, embryos were cleansed and incubated in the embryo medium (Danio-SprintM media) for two hours. Only healthy fertilised embryos were selected for the ZFET testing; meanwhile, the dead and coagulated embryos were discarded^35^.

### Zebrafish embryo toxicity (ZFET) test

Firstly, at 0 HPF, zebrafish embryos were exposed to samples (200 µL) in 96-well microplates (embryo/well) at seven different concentrations ranging from 0.16 to 10 mg/mL. The experiments were designed with an exposure group, both treated and untreated, containing 12 embryos each. The successfully treated embryos were cultured at ambient temperature (25 °C to 28 °C) for five days. The cumulative mortality and development abnormalities of zebrafish embryos and larvae were observed and examined for every 24 HPF from 0 to 120 HPF. Data of the survival rate, hatching rate, heart rate, morphological malformations, and teratogenic defects were captured and recorded using an inverted microscope coupled with a digital camera. The heartbeats were counted using a stopwatch (three embryos/min). Lethal endpoints were defined based on coagulation and the nonappearance of a heartbeat. Developmental defects such as pericardial oedema, yolk sac oedema, non-hatched, twisted body, and twisted tail were observed and recorded. The LC_50_ values were considered based on the principle of toxicity, in which > 1 mg/mL are considered relatively harmless, 0.1–1 mg/mL non-toxic, 0.01–0.1 mg/mL slightly toxic, 0.001–0.01 mg/mL moderately toxic, 0.0001–0.001 mg/mL highly toxic, and > 0.0001 mg/mL are super toxic.

### Ethics declaration

The breeding of Zebrafish (Danio rerio F. Hamilton, 1822) broodstocks and the in vivo methodology was approved by the Institutional Animal Care and Use Committee (IACUC) of Universiti Putra Malaysia (UPM), Malaysia and a licensed Danio Assay Laboratories Sdn. Bhd. (1,075,617-T), Director, Animal Biochemistry & Biotechnology Laboratory (ABBTech), Department of Biochemistry, Faculty of Biotechnology & Biomolecular Sciences, UPM, Selangor. The research was carried out in accordance with the Organization for Economic Cooperation and Development (OECD) No. 236: Fish Embryo Acute Toxicity (FET) Test (OECD, 2013)^56^, under compliance of IACUC UPM using triplicates of all samples and ARRIVE guidelines.

### Statistical evaluation

All of the graphs and figures were produced using GraphPad Prism v.8.0. (GraphPad Soft-ware, Inc.). The lethal concentration at 50% (LC_50_) of treated samples toward zebrafish embryos was evaluated using the same methods. The heart rates of three different animals were presented as a mean standard error of mean (SEM). A one-way analysis of variance (ANOVA) was used to determine significant differences, followed by a Dunnett's Multiple Comparison post-hoc test. differences between the means of the treated group and embryos in embryo media were set at *p* 0.001***, *p* 0.01**, *p* 0.05*.
